# Brain mechanisms of social signalling in live social interactions with autistic and neurotypical adults

**DOI:** 10.1038/s41598-023-46139-3

**Published:** 2023-11-01

**Authors:** Sujatha Krishnan-Barman, Uzair Hakim, Marchella Smith, Ilias Tachtsidis, Paola Pinti, Antonia F. de C Hamilton

**Affiliations:** 1 Institute of Cognitive Neuroscience, University College London, Alexandra House, 17 Queen Square, London, WC1N 3AR UK; 2 Department of Medical Physics and Biomedical Engineering, University College London, Gower Street, Malet Place Engineering Building, London, WC1E 6BT UK; 3 Centre for Brain and Cognitive Development, Department of Psychological Sciences, Birkbeck, University of London, Malet Street, London, WC1E 7HX UK

**Keywords:** Human behaviour, Cooperation

## Abstract

The simple act of watching another person can change a person’s behaviour in subtle but important ways; the individual being watched is now capable of signalling to the watcher, and may use this opportunity to communicate to the watcher. Recent data shows that people will spontaneously imitate more when being watched. Here, we examine the neural and cognitive mechanisms of being watched during spontaneous social imitation in autistic and neurotypical adults using fNIRS brain imaging. Participants (n = 44) took part in a block-moving task where they were instructed only to copy the block sequence which people normally do using a straight low action trajectory. Here, the demonstrator sometimes used an atypical ‘high’ action trajectory, giving participants the opportunity to spontaneously copy the high trajectory even if this slowed their performance. The confederate who demonstrated each block sequence could watch the participant’s actions or close her eyes, giving a factorial design with factors of trajectory (high/low) and watched (watched/unwatched). Throughout the task, brain signals were captured from bilateral temporal/parietal/occipital cortex using fNIRS. We found that all participants performed higher actions when being watched by the confederate than when not being watched, with no differences between autistic and neurotypical participants. The unwatched conditions were associated with higher activity of the right inferior parietal lobule in all participants and also engagement of left STS only in autistic participants. These findings are consistent with the claim that people engage different neural mechanisms when watched and unwatched and that participants with autism may engage additional brain mechanisms to match neurotypical behaviour and compensate for social difficulties. However, further studies will be needed to replicate these results in a larger sample of participants.

## Introduction

A starting point for social interaction is being in the presence of another person, a human who can watch, respond and communicate. The idea that ‘being watched’ changes behaviour is one of the oldest in psychology^1^ and has recently been the focus of new attention^2^. Measuring and understanding how both behaviour and brain activity change when being watched may help us understand the fundamental principles of social signalling^3^ and any differences in social signalling in autism. In the present paper, we examine how imitation behaviour changes when being watched. We consider these questions both in neurotypical and in autistic adults, and we use both motion tracking and wearable brain imaging to measure the cognitive processes involved in social imitation.

If a child and adult together place cutlery on the table for a meal, imitation learning may take place—the adult places the knife to the right of the plate, and the child learns to do the same. But social over-imitation can also occur—if the adult places the fork with a dramatic flourish, the child might do the same. This illustrates the distinction between two types of imitation: imitation to learn occurs when the child copies the adult’s goal-directed action (placing the knife), while social imitation occurs when the child copies the unnecessary flourish^4^. The latter is a spontaneous social behaviour which seems to be primarily about strengthening social connections between people. It has been described as ‘mimicry’^5^, the ‘chameleon effect’^6^ and a ‘social glue’^7^. This behaviour typically involves copying an action (or part of an action) that is not goal directed, such as action style^8^, the height of a pointing trajectory^9, 10^ or an irrelevant action in a sequence^11^. In typical adults and children, social imitation occurs more commonly when action goals are absent^10^ and when a participant is being watched^12, 13^.

**Figure 1:**
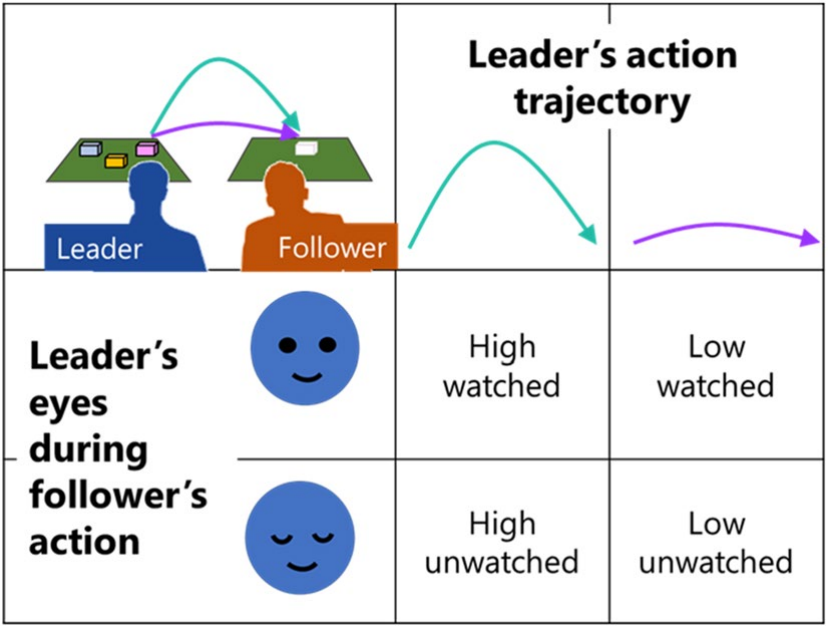
Factors studied. On each trial, the Leader moves a set of blocks and the follower is instructed to move the blocks in the same order. Copying action height is not mentioned. Trials can have high or low demonstrations from the Leader and the follower’s action can be watched or unwatched in a 2 × 2 design. Previous work shows more spontaneous social imitation of the high trajectories in the watched condition.

The case of ‘being watched’ is particularly important to the social signalling theory of imitation^3, 5^ and several studies suggest that being watched changes people’s behaviour. Participants imitate more when being watched by a video face^14, 15^, and direct gaze from a live actor enhances neural signatures of motor preparation^16, 17^. In both infants^18^ and children^13, 19–21^, more imitation behaviour is seen when being watched. The STORM theory provides a possible explanation of these data^5^. It suggests that imitation behaviour is used as a social signal to communicate to a partner “*I am like you”*, and that this signal will be sent primarily when the partner is watching, because there is no value in signalling to someone with their eyes shut^3^. The first direct test of this hypothesis was conducted by Krishnan-Barman et al.^12^. In an augmented-reality study, pairs of participants stood side-by-side to perform a block moving task, with one designated as leader and the other as follower. On each trial, the leader demonstrated a sequence of block movements and the follower was instructed *‘move the same blocks in the same order as quickly as possible’*. Sometimes the leader demonstrated a typical ‘low’ action trajectory in which they use a natural hand movement close to the table; while in other trials the leader demonstrated an atypical ‘high’ trajectory, moving high above the table as they shifted each block. Note that followers were never told to imitate movement trajectories, and in fact they would be more efficient in their actions if they did not imitate. This is because high trajectories have been described as irrational^22^ while low trajectories are closer to optimal for the fine control of the arm^23^ and allow for faster movements. Figure 1 illustrates this design. The results showed that when the leader had their eyes open during the follower’s turn, the follower showed stronger imitation of the ‘irrational’ high action trajectory, compared to a matched condition with the leader’s eyes closed. This demonstrates that people will spontaneously imitate action trajectories even with no instruction to do so, and that this behaviour is enhanced by the feeling of being watched. This result is in line with the claim that imitation behaviour can be used as a social signal to communicate with other people^3^. The present paper will use a very similar method to test if people with autism spontaneously imitate action trajectories and if their performance is affected by being watched.

Imitation has often been studied in autism, ranging from early studies^24^ to detailed reviews^25^ and examinations of brain mechanisms^26^. As early claims of a global mirror neuron dysfunction in autism^26^ seem unlikely to hold up^27^, there remains a gap in our understanding of whether there are any differences in how autistic and neurotypical people imitate actions. This matters because imitation provides a means to learn new skills and forge social connections^28^ and is a target for autism therapies^29^. In the brief review of imitation behaviour above, we distinguish between goal-directed imitation (e.g. moving blocks) and spontaneous social imitation (e.g. copying action trajectories). Increasing evidence suggests that spontaneous social imitation in particular is atypical in children and adults with autism. Autistic children do not copy the style of an action^8^ and do not overimitate unnecessary actions on objects^30^. Gaze and social engagement do not modulate imitation in those with autism spectrum conditions (ASC) to the same extent as in matched controls^31, 32^. Adults with autism do not imitate hand actions faster following a direct-gaze cue^33^ and nor modulate their action kinematics to match the demonstrator^10^. Responses to gaze and being watched may also be atypical in autism. Many studies report gaze-avoidance in autism^34^ but the effect of being-watched on spontaneous social imitation in adults with autism has not been systematically examined. Based on the social signalling theory^3^, we predict that adults with autism will show less spontaneous imitation of high action trajectories than typical adults and will not change their behaviour according to when they are being watched.

To further understand the cognitive processes supporting imitation behaviour, it is also informative to consider neural mechanisms. There is now increased focus on second person neuroscience^35^ and ecologically valid research^36^. In order to investigate imitation behavior in a naturalistic setting, we use functional near infrared spectroscopy (fNIRS) to capture the blood haemodynamic/oxygenation changes in the cortical surface^37^. fNIRS is silent, participant-friendly and robust to head, eye, face and body motion, so it is ideally suited to the study of imitation. It provides a substantial advantage over fMRI studies because participants are able to make hand and arm movements and engage socially with other people in the same room without the constraints of the MRI environment.

We will focus on parieto-temporal brain systems in our analysis of imitation mechanisms including the following regions: The inferior parietal lobule (IPL), which has a core role in human imitation behaviour^38^ as part of the mirror neuron system^39^. The left IPL is involved in observing and responding to goal-directed actions^40^, while the right IPL shows strong responses when participants observed actions with unusual trajectories^41, 42^ as used in this study. Temporoparietal junction (TPJ), which is strongly linked to theory of mind^43^ including the ability to interpret unusual actions^44^ and to the control of imitation behaviour^45^; Superior temporal sulcus (STS), which is engaged by a wide range of social stimuli including actions which seem irrational or which violate expectations^46–48^; Middle temporal gyrus (MTG) and lateral occipital cortex (LO), which have been linked to the perception of irrational actions^41, 49^ and the imitation of hand actions^50, 51^. These regions form a core network which is linked to imitation and social interaction, and are also accessible to fNIRS recordings.

We can draw specific predictions for engagement of right IPL and TPJ from a previous implementation of this task^52^. A study of 22 neurotypical adults used the design in Fig. 1 with fNIRS recordings from right temporal and parietal cortex in both participants. The most robust finding was less engagement of right IPL when followers perform a watched action compared to when they performed an unwatched action. That is, right IPL and TPJ were more engaged in the less social condition (unwatched) compared to the more social condition (watched). We interpret this result in terms of conflicting perspectives: in the unwatched condition there is a conflict between the participant’s own perspective (*I can see the blocks*) and the perspective of the leader (*He can’t see the blocks*), and processing this conflict might require more resources in IPL and TPJ. In the present study, we will use the same experimental design to extend our previous work, and test if the same pattern of results is found in a sample of neurotypical and autistic adults. Note that the present study is not a direct replication of our previous work because we have a different physical layout of objects and a different fNIRS device, but it is a conceptual replication.

Based on the papers reviewed above and our prior work, we can draw out specific predictions for the patterns of brain activation we expect to see. In neurotypical participants, we hope to replicate the results reported above, with less activation of right IPL and right TPJ in the ‘being watched’ conditions. In participants with autism, it is harder to make strong predictions because the neural mechanisms of imitation in autism remain controversial. While some studies report atypical engagement of parietal networks in autistic participants during imitation and action observation tasks^53, 54^, others report no differences^42^. Differences in activation of TPJ in autism have been reported in mentalising tasks^55–57^ but not by all groups^58^. The present study can add data from a naturalistic imitation task where detailed data for both performance and imitation are available. This is particularly important if we consider the possibility of *compensation* in people with autism^59^. Compensation describes the situation where people with autism show the same level of behavioural performance as those without autism, but might use different cognitive or neural mechanisms to do so. Our neuroimaging data will allow us to determine if participants with autism might be using different neural mechanisms to typical individuals in this imitation task.

### Summary of the present paper

The present paper builds on a recent task^12, 52^ in which a leader and a follower stand side by side and move blocks from one location to another following a specified order. Two factors are manipulated – does the leader move low (rational, ordinary action) or high (irrational unusual action) on each trial, and does the leader have her eyes open to watch the follower take a turn (watched condition) or does the leader closer her eyes (unwatched condition). These factors fall into a 2 × 2 factorial design (Fig. 1). The follower’s performance is quantified in terms of movement height, as high movements following a high demonstration from the leader indicate spontaneous imitation that was not specified in the instructions. Within this design, we can draw out four specific predictions based on the literature reviewed above. First, followers should copy the high action trajectories demonstrated by a leader, showing spontaneous social imitation. Second, if the follower imitates in order to *signal* to their partner, they should imitate more on trials where they are being watched. Third, the follower should be aware of whether they are being watched or not, reflected in increased activity in right IPL/TPJ when they are unwatched (due to conflicting perspectives). Fourth, both behaviour and brain activity patterns may differ in adults with autism, with less imitation and potentially different neural mechanisms engaged.

## Materials and methods

### Participants

Our target sample size (based on our previous work) was n = 20 participants per group. At the time the study was planned, there were no formal mechanisms available to implement a power calculation for fNIRS data analysis, so our target sample was based on roughly doubling the sample of our previous fNIRS study^52^ which had 22 participants. We recruited 25 neurotypicals (NT) and 26 autistic (ASC) participants using a local participant database, but seven participants (three NT and four ASC) were excluded from the analysis for failure to fully comply with task instructions. All seven participants failed to produce ‘high’ actions in the post-study trials when they were explicitly instructed to make these movements. This is similar to the exclusion rate in our previous studies^52^. Thus, the final analysis was conducted on 22 neurotypicals and 22 participants with ASC (see Table 1) which reflects the maximum availability of participants during the testing period.

**Table 1:** Participant characteristics.

	ASC (n = 22)		NT (n = 22)		p value for between-groups *t*-test
	Mean (SD)	Range	Mean (SD)	Range	
Age in years	33.8 (6.2)	21–45	30.2 (5.9)	19–39	0.06
Fullscale IQ	115 (13.9)	81–138	112.3 (14.4)	89–133	0.54
Verbal IQ	115.5 (14.7)	92–153	114.5 (12.3)	83–132	0.81
Autism quotient	32.2 (10.9)	10–47	16.1 (7.2)	7–33	< 0.001
Gender	5F; 17M			6F; 16M	
Handedness	1L; 21R			2L; 20R	

Groups were matched on gender, handedness, and on IQ using the Wechsler Adult Intelligence Scale versions III or IV (WAIS-IV, 2008, WAIS-III, 1997) ^60,61^ but differed on Autism Quotient^62^. Autistic participants had a diagnosis of Asperger’s syndrome (11), autism (5), or autism spectrum disorder (6), from an independent clinician and also completed module 4 of the Autism Diagnostic Observation Schedule^63, 64^ with a trained researcher who was independent of the study team. 11 participants met the ADOS classification for autism; 4 met classification for autism spectrum; 4 met classification on the communication subscale only; 2 did not meet classification and one had missing data. The ADOS scores in this group are consistent with recent studies which suggest that scores below cut-off are relatively common in highly intelligent adults^65–67^, and that such individuals are still autistic even if this is not detected on a standard ADOS. Given the clear and independent diagnostic history of all the autistic participants in our sample, we did not exclude any participants based on ADOS alone. All participants had normal or corrected-to-normal vision and hearing and had not participated in this experiment previously. Specific data on ethnicity, socioeconomic status and educational attainment levels were not recorded. Participants were reimbursed financially and provided informed written consent prior to participating. All procedures were approved by the UCL Graduate School Research Ethics Committee (Approval ID: 5975/003) and all experiments were performed in accordance with the relevant guidelines and regulations.

### Equipment

**Figure 2:**
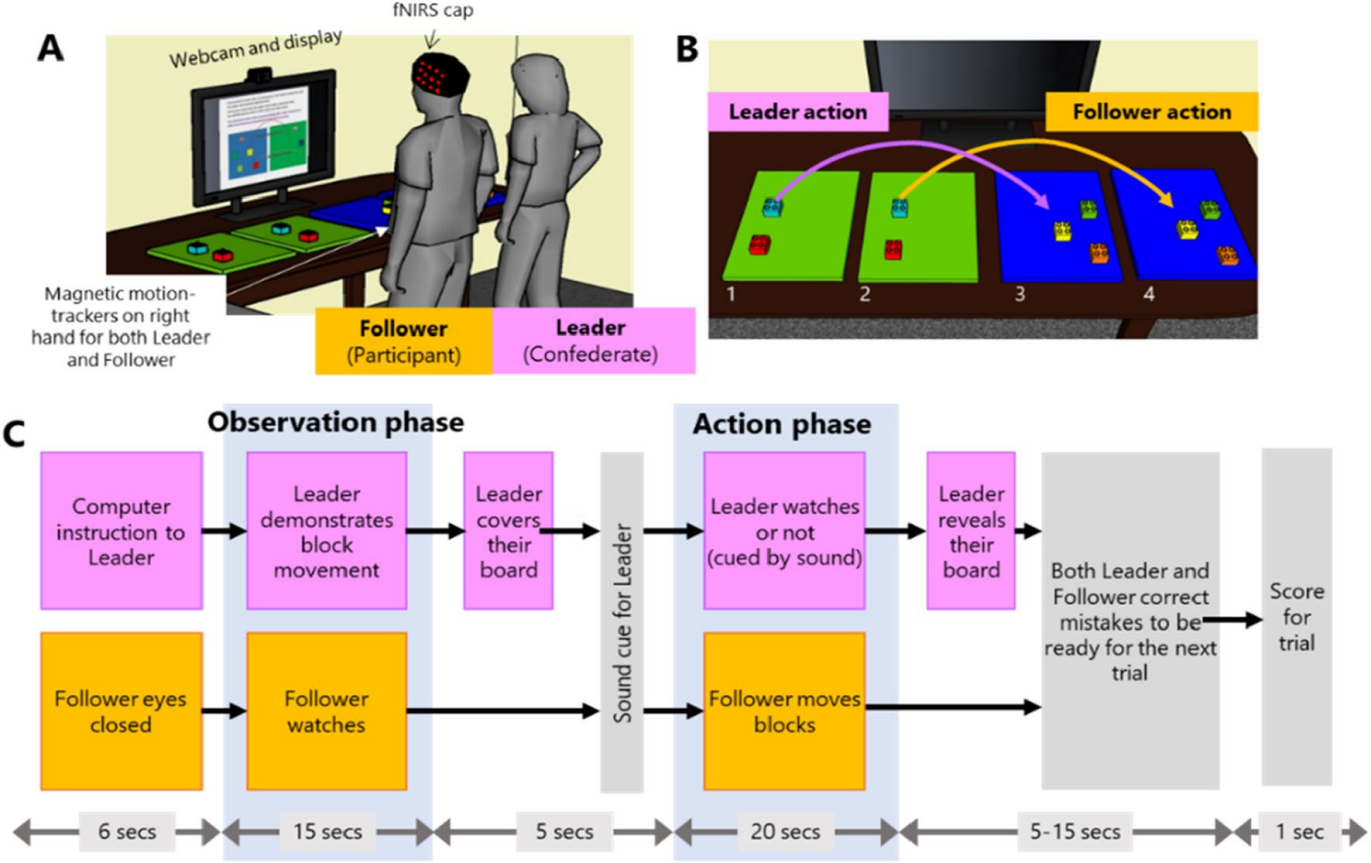
Methods. (**A**) An overview of the experimental setup. Figure shows the lab with the functional near-infrared spectroscopy (fNIRS) equipment. The Experimenter remained behind a curtain throughout the experiment. The leader was a trained confederate while the follower was a neurotypical or ASC participant. The follower’s brain activity was measured using the fNIRS equipment bilaterally centred on the temporal-parietal junction in each hemisphere. In addition to a Polhemus magnetic motion tracker, a webcam was also used to capture the social interaction. followers also wore a belt tracking heart rate, breathing rate and galvanic skin response. (**B**) Detail of the task space. Boards 1 and 3 were used by the leader who moved 3–4 Duplo bricks from one board to the other on each trial. The follower used boards 2 and 4, and was instructed to move the same blocks in the same order. The leader could use a high or low trajectory and the follower might spontaneously copy this trajectory. (**C**) Trial events and timings. Each trial begins with a computer instruction to the leader (not seen by the follower), then the leader demonstrates the movement sequence and covers the boards. A sound cue instructs the leader to close her eyes (or not) and then the follower moves his/her blocks. After the trial, the leader reveals her board and both people adjust the blocks to be ready for the next trial.

**Figure 3:**
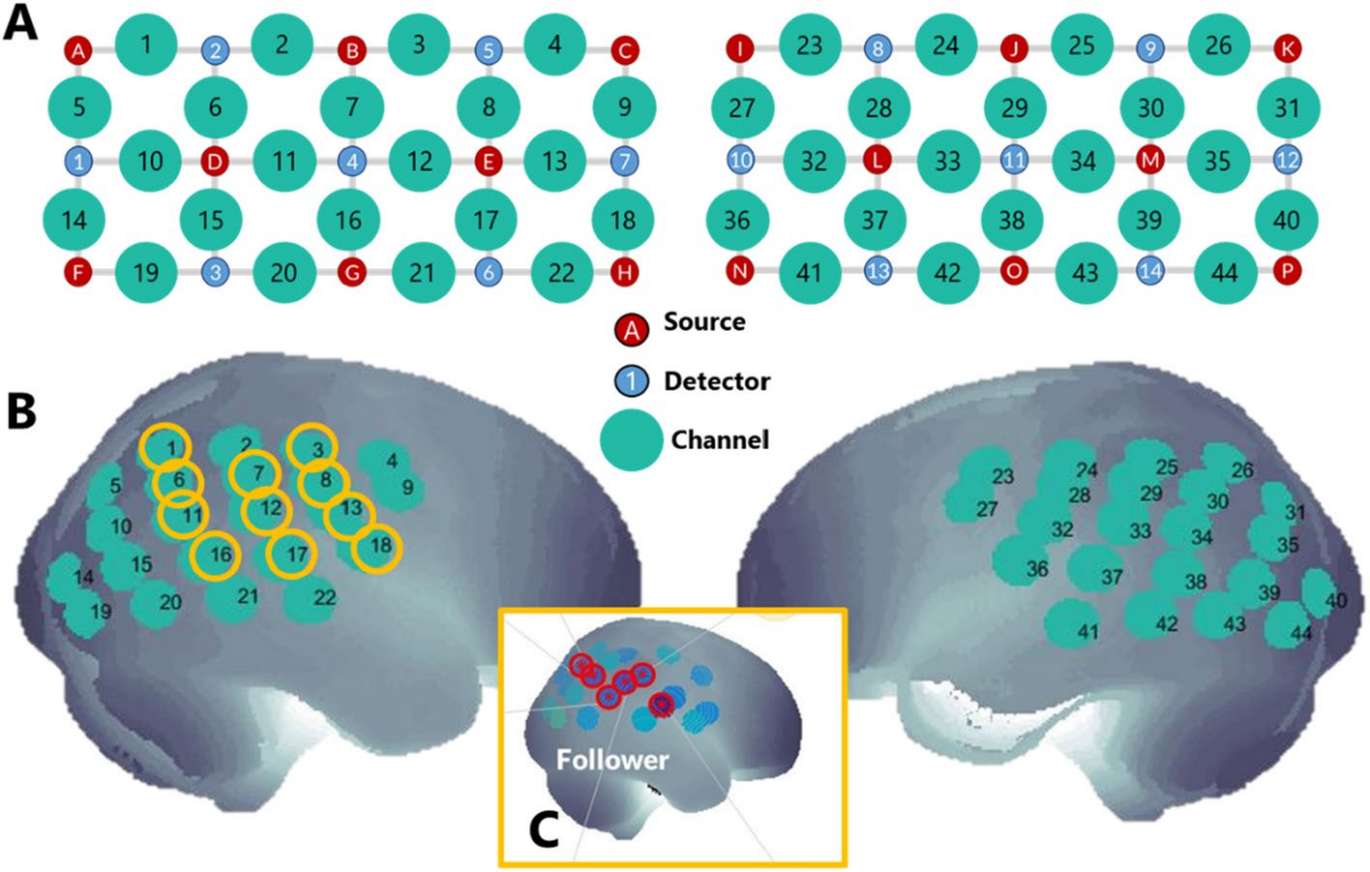
Optode configuration. (**A**) Diagram showing the positions of light sources (red), detectors (blue) and the 44 channels created by the 30 probes. (**B**) Locations of the 44 channels on the left and right hemisphere for each participant. Orange circles indicate the channels within the right parietal ROI. (**C**) Inset showing the results from the Watched > Unwatched contrast a very similar task (Krishnan-Barman 2021)^52^ used to define the ROI shown in (**B**).

The experimental room had a large desk with four Duplo baseplates and a set of coloured Duplo blocks (Fig. 2B) (Lego Company). These were arranged such that, on each trial, the leader (a confederate) could move a set of 3 or 4 blocks from one plate to another and the follower (the participant) could perform the same actions with the second set of plates and blocks. Plates 1 and 3 were used by the leader and boards 2 and 4 by the follower so both could clearly see each other’s actions. Both the participant and the confederate were standing throughout the study. A computer monitor provided instructions for the different task phases and a webcam recorded performance. A Polhemus Liberty magnetic motion tracker (Polhemus system, Colchester, Vermont) was used to capture behaviour during the task. This is a magnetic motion tracker which can capture precise marker locations without line-of-sight issues, so it is ideally suited to tracking finger trajectories in imitation tasks. Small markers were taped to the index finger of the leader and of the confederate and the locations of the markers relative to a fixed transmitter box on the desk were recorded at 240Hz throughout the experimental session. Physiological signals from the participant (ECG, respiration and galvanic skin response) were recorded with a wearable chest belt and finger electrodes (Equivital EQ02 Lifemonitor). These signals were included because it is best-practice in fNIRS studies to monitor systemic physiology^68^ but did not contribute to our overall hypotheses. Participants were fitted with an fNIRS cap to record hemodynamic signals from the cortex (LabNIRS, Shimadzu Corporation, Kyoto, Japan) including 30 optodes in a 44-channels array (Fig. 3). The LABNIRS system uses three wavelengths (780/805/830 nm) at a sampling frequency of 7.4Hz. A precise optode localisation procedure was used to ensure that cap placement and the locations of the recording sites in relation to the brain was consistent across participants, and this is detailed in the Supplementary Information.

### Procedure: preparation

When participants arrived for the experiment, they met the two experimenters and were told they would be participating in a team challenge with another participant who was a student at University College London (UCL). A young female confederate served as the ‘other participant’ for all data collection sessions. The confederate arrived after the participant and introduced herself to everyone, implying that she was unknown to the experimenters. Experimenter A assigned the role of leader to the confederate, and follower to the participant, and explained that the study was designed to look at how information is lost in movements. The leader was told to move blocks in an order demonstrated by the computer, and the follower was told to move the blocks in the order demonstrated by the leader, with a score given on each trial for quick and accurate movements. Note that these instructions emphasise fast and accurate copying of block order, which is best accomplished with low straight movements. Participants were not instructed to copy the height of the leader’s movement (movement height was never mentioned), and to copy this feature would make them slower and decrease the team score. They were asked to select a team name together and told that they would be competing against other teams. After the instructions, the recording equipment described above was fitted.

### Procedure: experimental tasks

The participant (follower) and confederate (leader) stood adjacent to each other, facing the table with the Duplo blocks (Fig. 2). Each trial had an Observation phase and an Action phase (Fig. 2C). First, the follower closed their eyes while the leader saw the block sequence and trajectory on the computer. As the leader was a confederate who was well-trained in the task, it was easy for the leader to implement the sequence of movements reliably. Then in the Observation phase, the follower watched the leader moving the blocks with either a low or high trajectory. After moving, the leader then covered their two boards with two cloths to prevent the follower seeing the block configuration and pressed the button to move the trial forward. The Action phase began with a sound cue instructing the leader to open or close her eyes; in the eyes-closed trials, the leader closed her eyes and put both hands over her face to make it very clear that she could not see. Then, the follower moved their blocks from one board to another, with the same order and locations that the leader had demonstrated. This took approximately 20 s. At the end of the trial, both participants corrected any errors in block location to prepare for the next trial, and the team received a score based on their speed and accuracy. The trials thus had a 2 × 2 factorial design with two different demonstration trajectories (low and high) and whether the Leader had their eyes open or closed during the follower’s turn (Watched and Unwatched). The four conditions were repeated four times over the 16 experimental trials, with trajectories presented in a randomised order. The Watched and Unwatched trials occurred in blocks of four trials; the initial block was randomised.

After the 16 experimental trials, participants completed a very short block of post-study control trials where the participant took the role of leader and was instructed to demonstrate high or low trajectories to the follower (confederate). Those who could not follow this instruction were excluded as detailed in the ‘participants’ section. Other data from these trials are not reported here. The complete fNIRS session took around 25–30 min per participant.

### Behavioural and physiological analysis

The main parameter used to evaluate imitative behaviour was the peak height reached by the follower on each trial. The Polhemus markers fixed to each person’s index finger record their precise location in centimetres relative to the Polhemus transmitter box on the desk. The data was segmented into trials and the maximum y-value (distance above the desk) was calculated as a simple measure of peak height on each trial. Participant’s peak heights on the post-study control trials were analysed to determine if they could perform ‘high’ movements when explicitly instructed to do so, and the 7 participants whose actions were the same on ‘high’ and ‘low’ control trials were excluded from all further analyses. For all participants included in the data, peak heights for each trial were analysed with an ANOVA with factors for diagnosis, demonstration height and whether or not the Leader was watching the trial. In all aspects of the data analysis (behaviour, physiology and fNIRS), we did not use any outlier detection method or exclude any outliers because this is not reliable for a small sample.

The Equivital belt recorded the participant’s ECG (256 Hz), respiration (25.6 Hz) and galvanic skin response (12.8 Hz). Each signal was filtered and converted to a simple rate (for heart and breathing, at 1 Hz) or GSR trace. The average heart-rate for each trial, average breathing for each trial and average GSR for each trial was calculated for each participant, and entered into an ANOVA to determine if physiological responses differed for the different trial types and participant groups.

### fNIRS analysis

Details of fNIRS preprocessing are given in Supplementary Information. After preprocessing, the data comprised 44 channels of fNIRS signals at 1Hz in which the oxy-Hb and deoxy-Hb data were combined using the correlation-based signal improvement method^69^ to give a single activation signal. Data for each participant was fit to a design matrix which modelled brain activation patterns in the follower with 6 regressors: Observation of high actions; Observation of low actions; Watched trials following high actions, Watched trials following low, Unwatched trials following high and Unwatched trials following low (Fig. 2C). Additional regressors for the instruction, cover, rearrange, score and constant phases of each trial were also included but physiological signals were not included in the GLM because they were not available for all participants. The first level design matrices were fit for each individual participant and each channel in SPM-NIRS^70, 71^, and the resulting betas were taken to the second level for contrasts analysis.

We evaluate three major contrast, related to our hypothesis. First, we localise activation in the follower during the observation of high actions compared to low actions, which captures brain systems responding to seeing an unusual action. Second, we localise activation during the action phase when the follower was being watched versus not being watched. This captures brain systems which are sensitive to the feeling of being watched. Third, we localise activation during the action phase which follows a high demonstration, compared to the action phase which follows a low demonstration. This captures brain systems engaged in responding to high actions (either by copying the action or ignoring it). Following the contrast calculations, *t*-tests were conducted on the beta values for each contrast in each valid channel separately for the neurotypical (NT) and the ASC groups. *t*-tests were also conducted to look for group differences between the two groups for each contrast, and to evaluate overall effects for both groups combined.

We evaluate our contrasts over the full optode array, and also evaluated only the Watched > Unwatched contrast within a region of interest based on our previous work. Briefly, Krishnan-Barman studied 22 neurotypical adults performing a task very similar to the present study and calculated a watched > unwatched contrast which engaged a cluster of 6 channels in right parietal cortex (Fig. 3C). Here, we created a ROI by assigning any channel within 1.5 cm of the 6 channels in the previous paper to our new ROI, giving 11 channels across right parietal cortex (Fig. 3B, orange circles). We highlight results which fall within this ROI and apply a Bonferroni correction for multiple comparisons to any results in this area. For results from other contrasts and those outside the ROI, we focus our discussion on results where the same pattern of data is seen in two (or more) adjacent channels as we believe these are more robust.

## Results

### Behaviour and physiology

**Figure 4:**
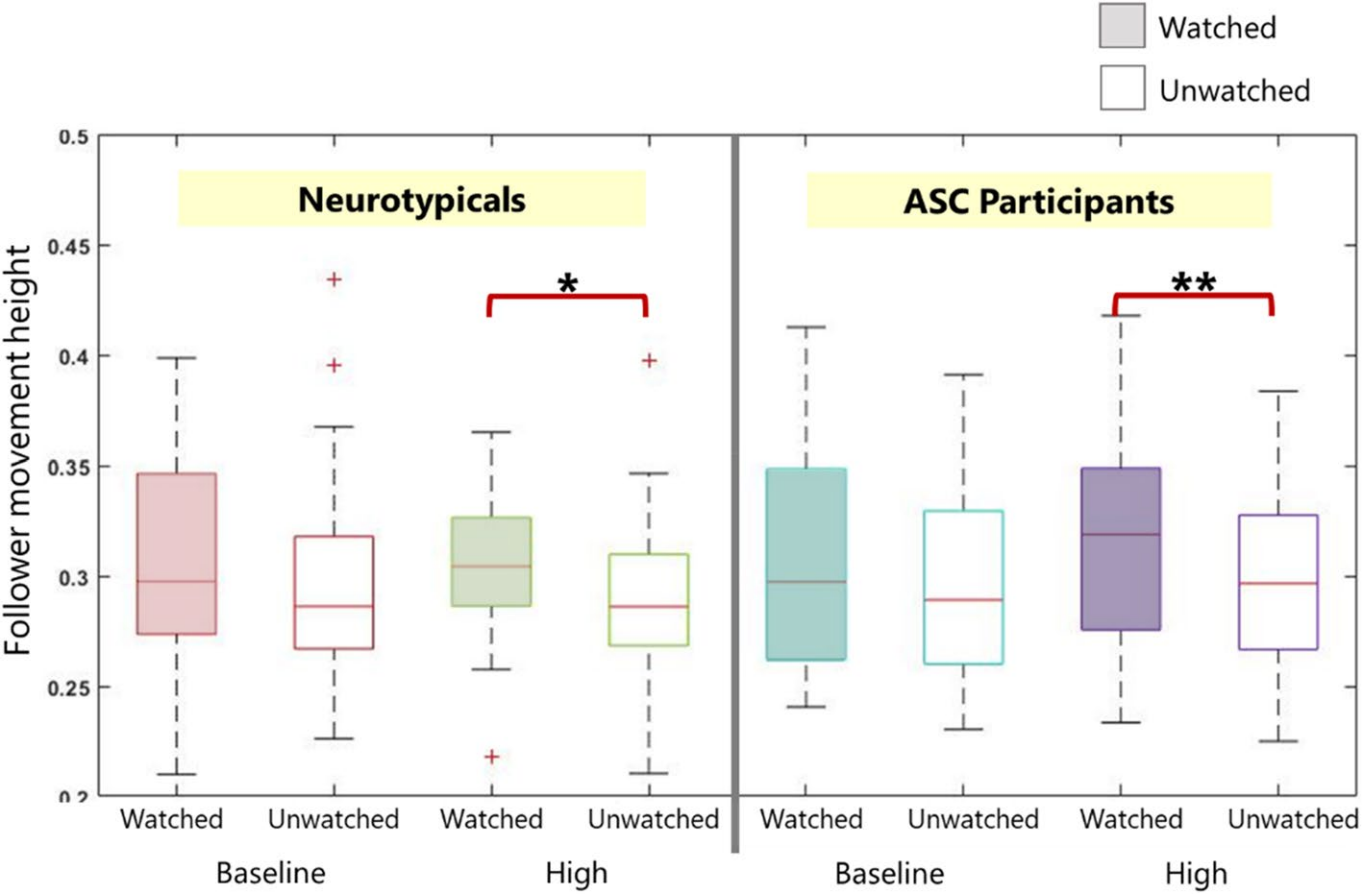
Three-way mixed ANOVA of follower height. This shows the height reached by the followers in both NT and ASC groups in the Baseline and High trajectory conditions, when being watched and unwatched. Paired-sample *t*-tests show significant differences in heights. *p < 0.05; **p < 0.01.

Behavioural analysis used a three-way mixed ANOVA compared the effect of being watched and of the leader’s trajectory (low or high) in both the NT and ASC groups and are shown in Fig. 4. We found a significant main effect of being watched on the follower’s movement height [F(1,42) = 7.29, p = 0.01]. We found no main effect of trajectory or group, and no significant two- or three-way interaction effects (full details in supplementary information). Subsequent paired-sample *t*-tests showed that participants in both NT and ASC groups reached a greater height in the Watched trials with High trajectory than in the Unwatched trials with High trajectory [NT: t(21) = 2.32, p = 0.03; ASC: t(21) = 2.86, p = 0.009]. This suggests that participants in both the NT and ASC groups moved with a higher trajectory only in trials where they were being watched.

Additional analyses of the time taken per trial showed that a significant main effect of being Watched on the time taken by the Follower [F(1,42) = 17.72, p < 0.001], with participants taking longer to complete trials when they were being watched. There was no main effect of Group or of Trajectory. There was a significant interaction effect between being Watched or Unwatched and Group [F(1,42) = 6.97, p = 0.012] (Supplementary Figure S2). A paired sample *t*-test showed that Autistic participants moved slower when being watched compared to the unwatched condition [M-Watched = 22.02, SD = 7.06, M-Unwatched = 17.26, SD = 5.26; t(21) = 4.22, p < 0.001]. Further conversation events during the trials, and the heart rate, breathing rate and GSR are in Supplementary Information.

### Neural mechanisms

**Figure 5:**
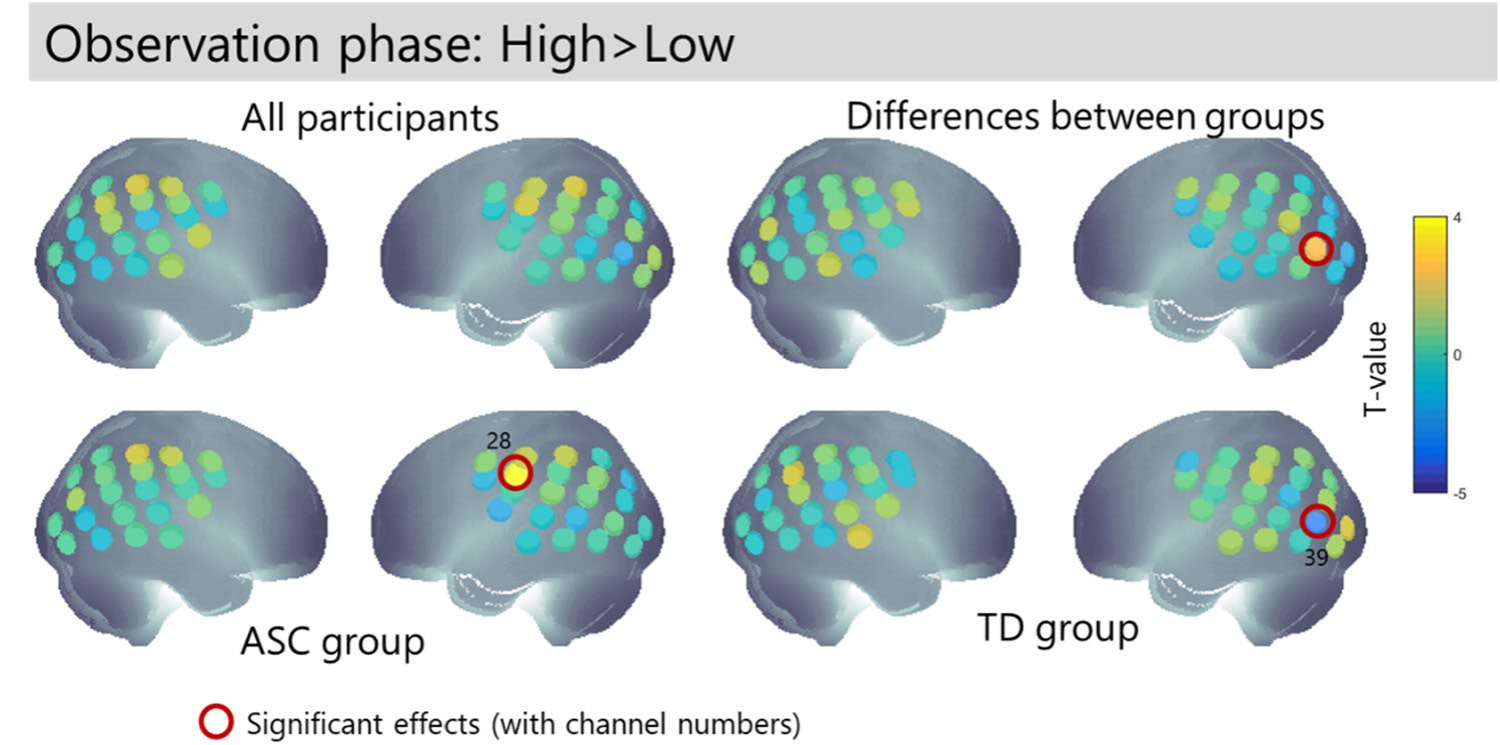
Follower’s brain activation when observing the leader perform High > Low actions. The T-values for the specified contrast are plotted at the channel locations on the canonical brain. Red circles indicate significant effects at p < 0.05 uncorrected.

Our analysis of neural mechanisms focuses on the three hypothesis driven contrasts presented above. In the Observation phase, followers saw the leader make a high or low movement; results are presented in Fig. 5. Left visual cortex (channel 39) showed less activation when NT participants observed High movements, and activation of this channel also differed between NT and ASC participants. Left somatosensory cortex (channel 28) was more activated when the ASC participants observed High movements. These results hint at differences in the observation of unusual actions between the typical and ASC participants, but all were isolated channels (Table 2) and did not meet corrected thresholds.

**Table 2:** Summary of neural activations for contrasts of interest.

Channel	Anatomical location	Observation phase: high > low		Action phase: watched > unwatched		Action phase after high > low	
		T	p	T	p	T	p
Significant effects in NT group							
2^a^	r IPL					− 2.47	0.04
31	l IPL			− 2.95	0.02		
39	l IPL	− 2.69	0.02				
44	l occipital					− 2.73	0.02
Significant effects in ASC group							
7^a^	r IPL			3.93*	0.001*		
24	l IPL					− 4.87	0.001
28	l PL	3.81	0.01				
37	STG			**2.93**	**0.01**		
41	MTG			**2.71**	**0.04**		
Significant differences between groups							
30	Angular gyrus					2.40	0.028
37	STG			2.774	0.013		
39	V3	2.2634	0.037			**2.33**	**0.032**
41	MTG					− 2.59	0.027
44	l occipital					**2.79**	**0.011**
Significant effects when groups are combined							
2^a^	r IPL			− **2.29**	**0.03**		
3^a^	r IPL			− **2.69**	**0.01**		
24	l IPL					− **4.07**	**0.001**
25	l IPL					− **2.17**	**0.04**
26	l IPL			− 2.64	0.02		

All effects which survive a p < 0.05 uncorrected threshold are listed. Channels marked with ^a^ fall within the a-priori ROI for the watched > unwatched contrast. Effects marked with * pass Bonferroni correction for multiple comparisons within the ROI. Effects in bold are found in two adjacent channels.

**Figure 6:**
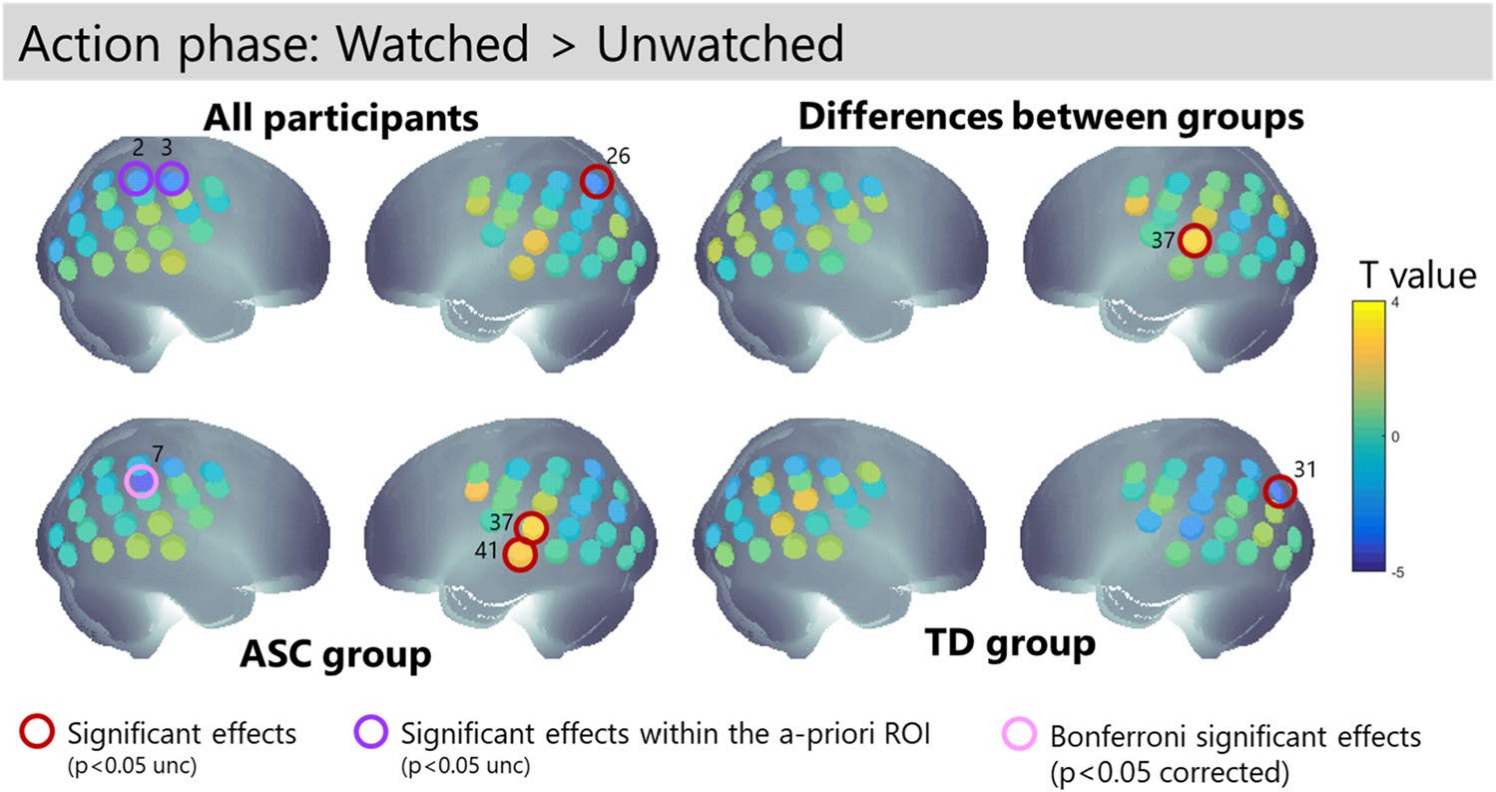
Channel-wise activation for the action phase of the task when participants are being watched by the follower. T-values are shown for each group and channels with p < 0.05 uncorrected are highlighted. Significant effects within the ROI are highlighted in purple.

During the action phase, participants performed the block moving task and could be watched by the leader or not (Fig. 6). This analysis was implemented both within our ROI (Fig. 3C) and over the whole array. Within the ROI, all participants engaged right parietal cortex (channels 2 and 3) when *not* being watched. In addition, autistic participants engaged right parietal cortex (channel 7) when *not* being watched and this effect met a Bonferroni correction for multiple comparisons within the ROI. Examination of the channels outside the ROI showed additional effects. In ASC participants, being-watched engaged regions of the left temporal cortex (channels 37 and 41), and one of these channels (37) showed a group difference between the NT and ASC groups with stronger activation in the ASC participants. There were also group similarities in the effect of being watched. However, none of these effects met corrected thresholds.

**Figure 7:**
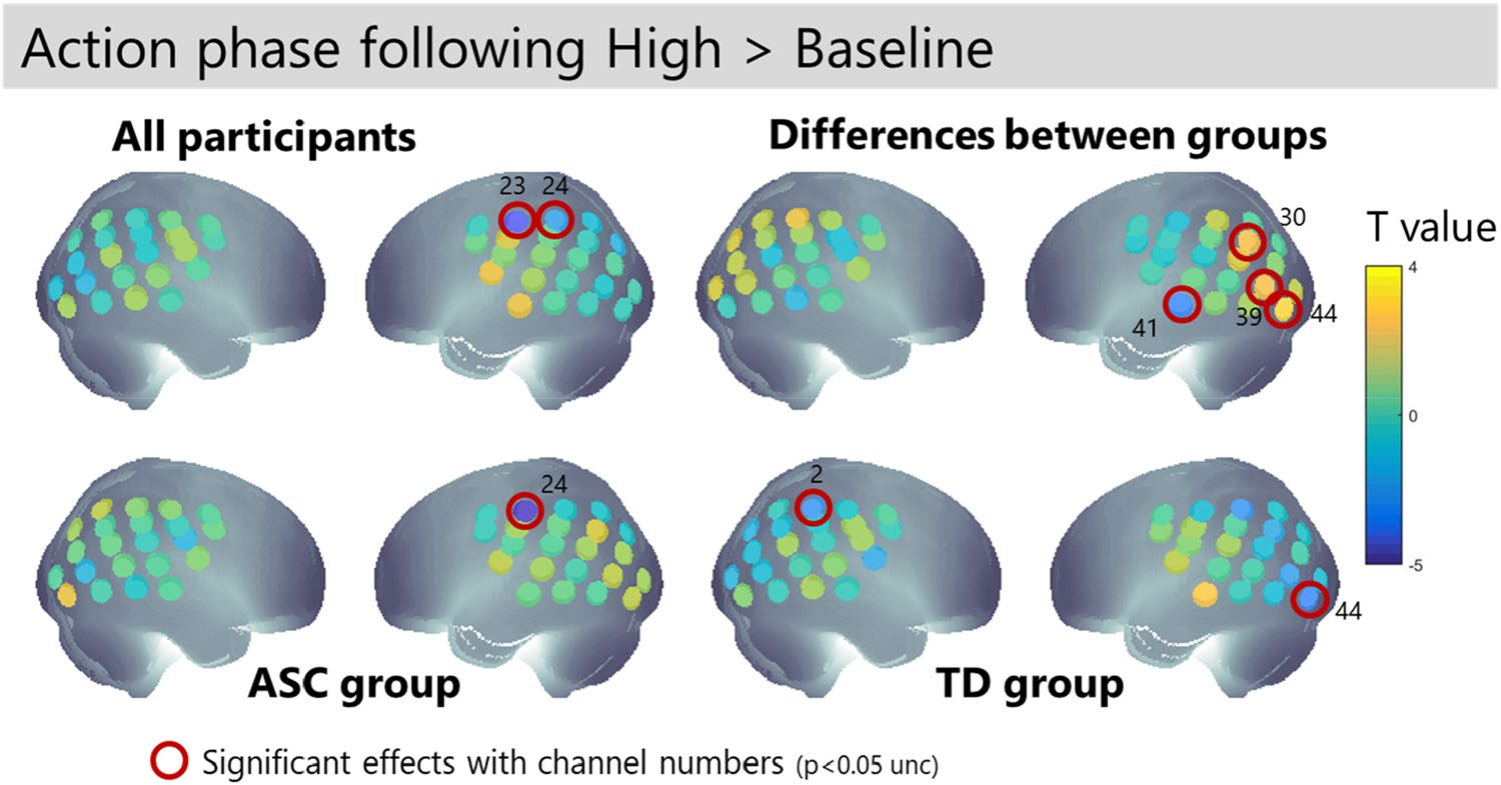
Channel-wise activation for the action phase of the task after a high demonstration by the leader, compared to a low demonstration by the leader. T-values are shown for each group and channels with p < 0.05 uncorrected are highlighted.

Comparison of neural activation during action phases that follow high versus low actions (Fig. 7) shows that both groups show suppression of left SMG extending to somatosensory cortex (channels 24 and 25) for the actions following high demonstrations. In addition, there are group differences in area V3 (channels 39 and 44), where the participants with autism show more engagement of this area than the neurotypical participants.

## Discussion

Changes in behaviour and brain activity when being watched provide a way to understand social signalling, and the present paper examines this in the context of imitation behaviour and autism. Using fNIRS in conjunction with an interactive task that enables spontaneous imitation of the height of action trajectories, we were able to test if participants with autism imitate, if their behaviour is modulated by being watched and what neural mechanisms might underlie this. We find comparable behaviour in typical and autistic adults: both groups spontaneously perform unusually high actions when being watched by the leader. Both groups engaged right parietal cortex in the Unwatched condition and this effect was most robust in the autism group. There were also group differences in neural activation patterns, as autistic participants engaged left STG and V3 regions substantially more than NT participants. We discuss these results in relation to the social-signalling theory of imitation and to the concept of neurocognitive compensation in autism.

### Being watched and imitation behaviour

In this study, we used a block-moving task where participants are asked to copy an action sequence to explore whether participants also spontaneously copy the height of the demonstrator’s trajectories. This task builds on extensive studies showing a distinction between goal directed imitation, in this case *copy the block order* and spontaneous social imitation, in this case of the *movement height*^4^. Participants were never instructed to copy movement height, and to do so would slow them down and impair performance. Nevertheless, both typical and autistic adults performed higher actions when being watched than when not being watched. This does not precisely replicate previous findings where participants imitated the movement height seen on each trial^9, 10, 12^, because we found no difference in movement height following observation of a high versus a low trajectory.

There are two possible explanations for this pattern of data. One is that it may reflect global imitation of high actions across the whole study. That is, we suggest the current task gave participants an overall context in which high actions might be a social norm and participants felt that they should make high actions, possibly without understanding why, and so did this more when being watched. We use the term ‘global imitation’ to reflect the idea that, if our whole study had not contained high actions, then participants would probably not have produced high actions, so we suggest they were imitating an overall social norm set by the experimental context. Many developmental studies show children perceive and imitate social norms^72, 73^ and the same could apply here. Unfortunately, the trial ordering did not allow us to explicitly ask participants if they noticed that some actions were high or if they imitated on purpose, so we do not have data on why participants showed this pattern of behaviour.

A second explanation could be that the pattern of moving higher when being watched reflects a general audience effect, and an increase in communicative behaviour in this condition^74^. This explanation assumes there is nothing specific about the task context or high actions of the demonstrate that causes high actions from the participant, and only the effect of being watched matters. A study in which demonstrators performed different types of unusual actions, for example moving high on some trials and curving away from their body on other trials, might be needed to distinguish between these options. However, both explanations can be clearly linked to ideas about social signalling and socio-motor communication—both assume that behaviour changes when being watched, and we find evidence for this.

There are several differences between our design and previous work^10, 12, 52^ which could explain why we did not see clear trial-by-trial imitation behaviour in this study. Previous studies used pointing movements as participants watched videos^10^ or moved in an augmented reality space^12^ whereas in the present study, participants moved real physical blocks. Also, previous studies used either video stimuli or pairs of naïve participants^12^ to provide model trajectories; for practical reasons the current study used a confederate which can be problematic^75^. Given these factors, we cannot draw strong conclusions about why participants showed a global imitation of action height but not a trial-specific pattern of imitation in this task.

Despite this, we do show a clear effect of being watched on the height of the movements performed by participants: movements were higher when being watched. This is in line with other recent work that has shown that whether an interaction partner is watching us or not has an impact on a wide range of behaviours including the fidelity with which we imitate^13, 19–21^. Here, we found that this effect is preserved in autistic participants who also performed more high actions when being watched. Increased global imitation when participants are being watched is consistent with the social signalling hypothesis^3^ and with the idea that people often imitate or conform to norms in order to send signals to other people. We predicted that autistic people might engage less in social signalling and show less spontaneous imitation when being watched^3^, but we did not find this effect. This could reflect compensation in our participant sample, where participants with autism are able to act as if they are neurotypical, but possibly using different cognitive mechanisms. Based on our study design it is not possible to parse whether autistic participants engaged in this social imitation deliberately, because we could not question the participants on whether they detected the ‘high’ actions as an unusual behaviour without disrupting performance. Thus, conclusions about participant awareness of the trajectory height manipulation in this and similar studies will have to await further research.

Other studies of audience effects have suggested that differences in behaviour may result from differences in arousal from direct gaze^76^, differences in anxiety^77^ or simple social facilitation^78^. Our study was designed to minimise the differences between the watched and unwatched condition: participants were side-by-side with no differences in eye-gaze or in social facilitation between conditions. Further, the null result for galvanic skin response between the watched and unwatched condition (Supplementary Info) supports the view that there was no difference in arousal. This buttresses our claim that the behavioural differences seen between the watched and the unwatched condition arise from the cognitive effect of being watched, rather than explanations rooted in arousal or social facilitation.

### Brain mechanisms of social signalling and imitation

Behaviourally, being watched impacted on action trajectories in both neurotypical and autistic participants, and the fNIRS neuroimaging data also shows effects. Across both groups, there was less activation in the right IPL on trials when participants were being watched. This result directly replicates our previous study^52^, and in the autism group this finding met our strict correction for multiple comparisons. The striking finding that right IPL is less active on ‘being watched’ trials could not be predicted from previous fMRI studies, where it is hard to create a sense of being watched or to enable participants to engage in spontaneous imitation behaviour. Based on prior literature, one might have expected stronger engagement of right IPL and right TPJ in the more ‘social’ condition of being watched, but there are several possible reasons for the pattern of results we see.

One possibility is that reduced IPL engagement when being watched could be related to participant’s motor behaviour, as they moved higher (and thus copied closely) in the watched condition. If imitation is a ‘default’ for the motor system, simple imitation might not require much resources. In the unwatched condition, if participants do not imitate the demonstrator’s action they might need to devote more cognitive resources to motor planning for their own movement, which would lead to more activation of IPL^79^. An alternative explanation could be due to the perspective taking requirements of the task. We know that people can spontaneously take the perspectives of others^80^ and that tracking different perspectives demands resources in TPJ and IPL^81^. When standing next to another person, it would be natural to assume that the other person has the same perspective as oneself with no conflict. However, in our Unwatched condition, the other person in the room has closed her eyes creating a conflict between the participant’s view (*I can see everything*) and the confederate’s view (*She can’t see*). Tracking this conflict could demand more engagement of right IPL and TPJ, leading to greater brain activity in the unwatched conditions. The present data cannot distinguish between these possibilities, but it would be important to do so in future studies.

This study also examined brain activity when watching high or low actions and performing high or low actions. We found only minimal brain effects of observation of high or low actions, but there were differences when participant took their turn after the observation of high or low actions. Across both ASC and NT participants, activation of the left IPL was reduced for trials following high actions compared to low actions. This is an unexpected result, as we might expect a larger slower high action to result in a greater activation in motor regions of the brain. However, there are few precedents for this type of result, because fMRI studies do not permit large scale spontaneous hand and arm movements during scanning, and there are few prior fNIRS studies of motor performance. Thus, more research on naturalistic motor actions in conjunction with fNIRS brain imaging will be very valuable.

### Differences between neurotypical and autistic participants

We found possible differences between the NT and ASC groups in the left lateral occipital cortex when performing an action after viewing a high or low action, with greater activation in the ASC participants than the NT participants. This activation pattern is potentially robust because it was found in two adjacent channels, however, we could not use an ROI because there was no prior data for this contrast. While lateral occipital regions are not traditionally considered part of the human MNS, there is strong evidence that these brain areas are engaged in matching the actions of self and other^82^ and in imitation tasks^50^, particularly delayed imitation as used here^51^. We also found differences between the NT and ASC participants in our examination of ‘being-watched effects’. Participants with autism showed more activity in left STS when being watched compared to not being watched, and these same channels also showed a group difference with greater activity than the same channels in the NT participants. This part of left STS is not strongly associated with imitation, but is linked to social perception and social interaction^83^.

To summarise, two major contrasts show greater activation in autistic participants than in neurotypical participants during a social imitation task, with both clusters located in the left temporal or occipital cortex. Prior studies have suggested that participants with autism might show reduced imitation behaviour on this type of task^10, 30^ but we did not find behavioural differences here. Thus, we suggest that the stronger brain activation patterns in ASC participants might reflect compensation^59^ and the recruitment of additional neurocognitive resources in order to achieve stronger behavioural performance. This signposts interesting differences in neurotypical and autistic cognitive processes despite similar behavioural responses. However, as these results did not meet a strict correction for multiple comparisons, they must be considered indicative at this stage and further studies will be needed.

### Limitations

There are many challenges to building a naturalistic paradigm which will allow participants to engage in spontaneous social imitation while still being able to record neuroimaging data. Our block-moving task controls whether the participant’s action is watched or not, but does not measure participant’s awareness of the high/low trajectories they saw or potential deliberate strategies. This is because asking participants questions about their perceptions and their strategy might change their behaviour. Furthermore, this task which involved moving physical blocks is not quite the same as previous tasks involving pointing actions^9, 10, 12^, and the patterns of behaviour also did not quite replicate previous work. Here, we found a global tendency to make higher movements when being watched, rather than a precise trial-by-trial pattern of imitation. This might reflect differences in the goal-directedness of the actions, because social imitation effects can be stronger with no goal^10^ or might reflect social norm setting in the current task or might reflect a general audience effect; further study would be needed to distinguish these. Because the behavioural effects in both the autism and neurotypical groups did not match our predictions, we cannot draw strong conclusions about imitation behaviour in participants with autism.

A further limitation concerns the issues of statistical power and correcting for multiple comparisons in fNIRS data. We are not aware of any ways to calculate a priori statistical power for fNIRS studies in order to plan sample sizes or trial numbers, and did not have access to any way to do this when the study was planned. Thus, we chose a design which fitted as many trials as possible into the available time (fNIRS caps become uncomfortable after 25 min of use) with a sample size that nearly doubled our previous study^52^. From the data above, it seems likely that this design is underpowered and future studies with more trials and larger samples would be valuable. The best ways to obtain robust corrections for multiple comparisons in fNIRS data is also an underexplored topic. For our Watched > Unwatched contrast, we used an a priori ROI from a previous very similar study to constrain our tests and applied Bonferroni correction within this ROI. Here, we find a robust result in the autism group and indications of a similar effect in the neurotypical group. The fact that we find suppression of right IPL when being watched twice in two independent samples adds strength to our data. For other contrasts and regions outside this ROI, we report uncorrected data and focus our discussion on data where pairs of adjacent channels pass the p < 0.05 threshold, as these are less likely to occur by chance. Nevertheless, the present data should be considered indicative rather than definitive and further replication and exploration will be valuable.

Finally, the group of participants who volunteer to take part in our research are probably not representative of the whole autism population. While many people with autism have limited verbal skills and substantial support needs, our participants have IQ scores in the normal range and are able to take part in complex brain imaging research studies. This means that the results we find may not generalise to the whole autistic population. We note that the same is true of most studies using functional neuroimaging to study autism, and that fNIRS has the potential to be used with a wider range of participants as it is silent and wearable and is often considered less stressful than MRI. Thus, it would be interesting to implement similar studies in future with a more varied group of participants in future, including children with and without autism.

### Future directions

This study opens the way to a range of future research. Here, we demonstrate that it is possible and valuable to study natural spontaneous imitation behaviour using fNIRS, and that the simple manipulation of ‘being watched’ can have substantial effects on both behaviour and brain activation patterns. Future replications of this approach would benefit from adding eyetracking systems to capture participant’s gaze behaviour during the task. More broadly, our results support the claim that movement height can be used as a social signal, that is, people perform unusual actions when they can be seen by an interaction partner in order to communicate with their partner. Tracking the generality of this effect across different social contexts and how it varies in autism will be useful in future.

### Supplementary Information

Supplementary Information.

## Data Availability

Data from this project is available at https://osf.io/zc73w/?view_only=6c07a617bbcb4b28b8f4575e04b6328a.
