# Supplementary material for: Brain mechanisms of social signalling in live social interactions with autistic and neurotypical adults
Source: Sci Rep. 2023 Nov 1;13:18850. doi: 10.1038/s41598-023-46139-3 (PMC10620418; doi:10.1038/s41598-023-46139-3)
Supplement: Supplementary file 1 — Supplementary Information. [file 41598_2023_46139_MOESM1_ESM.pdf]

Supplementary Information for Krishnan-Barman et al.

fNIRS optode localization

A spandex cap fitted with a rigid probe holder was used to hold the optodes, allowing us to fit participants with varying head sizes, while keeping a source-detector distance of 3cm. To obtain accurate localisation of the optodes on each participant's head, we used custom software in conjunction with a 3D Magnetic Digitizer (Polhemus Liberty) equipped with 4 motion sensors. One large sensor was taped to the participant's forehead to correct for head displacement respect to the magnetic field source during the recording, while a second sensor was used to capture the location of the five canonical head locations (inion, nasion, right preauricular, left preauricular and vertex) and each optode. Custom Matlab software was used to calculate the location of each point relevant to the head-fixed sensor, thus providing precise localisation that are corrected for any head motion during the capture session. SPM-fNIRS (Tak et al., 2016; Tak and Ye, 2013) was used coregister the captured points onto a brain model and obtain MNI coordinates for each optode. Supplementary Table S1 lists the mean MNI coordinates and likely anatomical localisation of all optodes.

Supplementary Table 1: Channel coordinates and anatomical regions. Channels are listed below alongside their Montreal Neurological Institute (MNI) coordinates (Mazziotta et al., 2001). The corresponding anatomical region labels are obtained from the NIRS-SPM software (Ye et al., 2009) based on the included atlas (Rorden & Brett, 2000). Alongside each anatomical region the corresponding Brodmann Area (BA) and the probability that this region is included in the channel are shown.

Channel number	MNI coordinates X	Y	Z	Anatomical region	BA	Probability of inclusion
1	48.3	-66.9	49.3	Angular gyrus, part of Wernicke's area	39	0.85
1				Somatosensory Association Cortex	7	0.13
2	60.5	-44.7	50.3	Supramarginal gyrus part of Wernicke's area	40	1.00
3	63.4	-23.4	48.8	Primary Somatosensory Cortex	1	0.55
3				Primary Somatosensory Cortex	3	0.21
3				Supramarginal gyrus part of Wernicke's area	40	0.12
3				Primary Somatosensory Cortex	2	0.06
3				Primary Motor Cortex	4	0.06
4	59.0	1.0	45.3	Pre-motor and Supplementary Motor cortex	6	0.80
4				Primary Motor Cortex	4	0.18
5	37.6	-84.2	36.4	V3	19	0.93
5				Angular gyrus, part of Wernicke's area	39	0.05
6	56.2	-65.1	38.3	Angular gyrus, part of Wernicke's area	39	1.00
7	65.7	-41.9	40.8	Supramarginal gyrus part of Wernicke's area	40	0.98
8	67.5	-19.3	38.8	Primary Somatosensory Cortex	1	0.44
8				Primary Somatosensory Cortex	2	0.40
8				Subcentral area	43	0.09
8				Primary Somatosensory Cortex	3	0.07

Channel number	MNI coordinates X	Y	Z	Anatomical region	BA	Probability of inclusion
9	62.6	4.5	36.1	Pre-motor and Supplementary Motor cortex	6	0.67
9				Primary Motor Cortex	4	0.16
9				Subcentral area	43	0.12
9				pars opercularis, part of Broca's area	44	0.05
10	46.5	-83.2	23.0	V3	19	0.74
10				Angular gyrus, part of Wernicke's area	39	0.26
11	61.5	-61.4	27.0	Angular gyrus, part of Wernicke's area	39	0.60
11				Superior Temporal Gyrus	22	0.35
12	69.0	-39.0	29.2	Supramarginal gyrus part of Wernicke's area	40	0.50
12				Superior Temporal Gyrus	22	0.23
12				Retrosubicular area	48	0.22
12				Primary Somatosensory Cortex	2	0.06
13	69.3	-14.7	29.7	Subcentral area	43	0.49
13				Primary Somatosensory Cortex	2	0.40
13				Primary Somatosensory Cortex	1	0.10
14	35.3	-96.8	6.2	Visual Association Cortex (V2)	18	0.70
14				Primary Visual Cortex (V1)	17	0.26
15	53.9	-77.4	10.4	V3	19	0.66
15				Fusiform gyrus	37	0.22
15				Angular gyrus, part of Wernicke's area	39	0.12
16	67.0	-53.9	13.6	Superior Temporal Gyrus	22	0.35
16				Middle Temporal gyrus	21	0.34
16				Fusiform gyrus	37	0.31
17	71.6	-31.3	13.8	Superior Temporal Gyrus	22	0.89
18	69.4	-6.7	17.0	Subcentral area	43	0.60
18				Superior Temporal Gyrus	22	0.31
18				Retrosubicular area	48	0.08
19	44.6	-89.4	-5.1	V3	19	0.61
19				Visual Association Cortex (V2)	18	0.39
20	59.8	-68.2	-2.1	Fusiform gyrus	37	0.90
20				V3	19	0.10
21	70.7	-45.6	-0.2	Middle Temporal gyrus	21	0.43
21				Superior Temporal Gyrus	22	0.26
21				Fusiform gyrus	37	0.18
21				Inferior Temporal gyrus	20	0.14
22	72.6	-22.9	-0.8	Middle Temporal gyrus	21	0.71
22				Superior Temporal Gyrus	22	0.29
23	-55.0	1.8	46.3	Pre-motor and Supplementary Motor cortex	6	0.87
23				Primary Motor Cortex	4	0.11
24	-60.5	-23.0	48.7	Primary Somatosensory Cortex	3	0.40
24				Primary Somatosensory Cortex	1	0.30
24				Primary Somatosensory Cortex	2	0.17
24				Primary Motor Cortex	4	0.07
24				Supramarginal gyrus part of Wernicke's area	40	0.07
25	-58.6	-46.6	49.3	Supramarginal gyrus part of Wernicke's area	40	0.99
26	-47.8	-68.6	48.2	Angular gyrus, part of Wernicke's area	39	0.90
26				Somatosensory Association Cortex	7	0.08
27	-59.0	6.6	35.5	Pre-motor and Supplementary Motor cortex	6	0.68
27				pars opercularis, part of Broca's area	44	0.18
27				Primary Motor Cortex	4	0.11
28	-64.6	-18.2	39.1	Primary Somatosensory Cortex	1	0.38

Channel number	MNI coordinates X	Y	Z	Anatomical region	BA	Probability of inclusion
28				Primary Somatosensory Cortex	2	0.33
28				Primary Somatosensory Cortex	3	0.17
28				Subcentral area	43	0.13
29	-64.3	-42.8	39.3	Supramarginal gyrus part of Wernicke's area	40	0.92
29				Retrosubicular area	48	0.08
30	-56.0	-66.0	36.6	Angular gyrus, part of Wernicke's area	39	1.00
31	-39.2	-85.5	33.8	V3	19	0.92
31				Angular gyrus, part of Wernicke's area	39	0.08
32	-67.2	-14.7	27.9	Subcentral area	43	0.48
32				Primary Somatosensory Cortex	2	0.31
32				Retrosubicular area	48	0.11
32				Primary Somatosensory Cortex	1	0.07
33	-67.4	-39.5	27.5	Retrosubicular area	48	0.34
33				Superior Temporal Gyrus	22	0.31
33				Supramarginal gyrus part of Wernicke's area	40	0.28
33				Primary Somatosensory Cortex	2	0.06
34	-61.6	-60.7	24.8	Angular gyrus, part of Wernicke's area	39	0.50
34				Superior Temporal Gyrus	22	0.31
34				Fusiform gyrus	37	0.08
34				Middle Temporal gyrus	21	0.06
35	-47.5	-83.3	22.4	V3	19	0.77
35				Angular gyrus, part of Wernicke's area	39	0.23
36	-66.6	-6.9	14.3	Subcentral area	43	0.41
36				Superior Temporal Gyrus	22	0.40
36				Retrosubicular area	48	0.18
37	-69.3	-31.2	12.2	Superior Temporal Gyrus	22	0.85
37				Middle Temporal gyrus	21	0.06
37				Primary and Auditory Association Cortex	42	0.06
38	-66.1	-54.2	10.9	Fusiform gyrus	37	0.38
38				Middle Temporal gyrus	21	0.37
38				Superior Temporal Gyrus	22	0.26
39	-54.8	-76.4	7.7	V3	19	0.68
39				Fusiform gyrus	37	0.29
40	-37.1	-96.1	4.9	Visual Association Cortex (V2)	18	0.85
40				Primary Visual Cortex (V1)	17	0.11
40				V3	19	0.05
41	-70.4	-23.7	-5.7	Middle Temporal gyrus	21	0.94
41				Superior Temporal Gyrus	22	0.06
42	-68.6	-46.1	-3.0	Middle Temporal gyrus	21	0.41
42				Fusiform gyrus	37	0.24
42				Inferior Temporal gyrus	20	0.24
42				Superior Temporal Gyrus	22	0.11
43	-59.7	-67.7	-3.8	Fusiform gyrus	37	0.95
43				V3	19	0.05
44	-45.1	-88.5	-6.3	V3	19	0.78
44				Visual Association Cortex (V2)	18	0.22

fNIRS preprocessing

The LabNIRS captures light intensity at 3 wavelengths (780 / 805 / 830nm) from 16 detectors at 7.4 Hz throughout the experiment. These data were first converted to changes in optical density and then into concentration changes of oxy-Hb and deoxy-Hb in 44 channels by the modified Beer-Lambert law with a fixed Differential Pathlength Factor (DPF) of 6. Preprocessing data was performing using HomER2 (Huppert et al., 2009). The data for each of the 44 channels across 44 valid participants was examined as follows. First, we visually inspected the Power Spectral Density (PSD) for each participant at each channel to look for a peak between 1-2Hz corresponding to the heartbeat oscillation. A lack of a heartbeat oscillation could suggest that the optical coupling between the optode and the scalp was poor and hence no brain signal was being measured (Hernandez and Pollonini, 2020). Second, the raw intensity data was inspected for detectors' saturation and artefacts. Third, the oxy-Hb and deoxy-Hb signals were inspected to see if they were positively correlated (the hemodynamic response should show a negatively correlated oxy-Hb and deoxy-Hb with deoxy-Hb having 2-3 times smaller magnitude than oxy-Hb (Tachtsidis and Scholkmann, 2016). Based on these criteria individual channels were excluded. If any participant had more than 50% of the channels in one hemisphere excluded, that hemisphere alone was excluded from the analysis. A total of six participants were excluded entirely, largely owing to them having very thick hair or fixed hairstyles which prevented optimal optode-skin coupling.

For all channels which passed the signal quality checks, data was motion corrected using a Wavelet based motion correction method (Molavi and Dumont, 2012) with the default iqr value of 1.5. In line with the recommendations made by Pinti et al. (2019), we applied a bandpass filter (0.01 Hz to 0.3 Hz) to denoise the data. In the initial stage of the experiment, we used a slightly different configuration for the optodes, including the PFC and two 3x4 arrays of optodes over the bilateral TPJ. However, two out of the three participants who had this configuration were unable to complete the experiment due to discomfort in the forehead. We therefore dropped the PFC and used a configuration involving two 3x5 arrays as described above. These three participants were also excluded from the fNIRS analysis, bring the total number of participants whose neural signals were analysed to 35.

Normalising channel locations for cross-participant fNIRS data is often a challenge, especially for lateral regions of the cortex where small differences in head size can have a large impact on optode location with respect to the brain. Since we had digitized the optode locations for participants wherever possible, we were able to plot the actual positions of the channels for all participants as well as the canonical average channel centres. These data (Supplementary Figure 1) allowed us to check if the consistency of the channel locations across our group of participants and to ensure that we only average data across participants if it comes from the same cortical location. We found that the average distance between adjacent canonical channel centres was 20.8mm. Based on this, we set a threshold of 10mm and excluded any channels that were more than 10mm away from their corresponding channel centre. Out of 1,284 valid channels available for analysis, 909 channels survived this thresholding (Supplementary Figure 1B) and these are analysed below.

Supplementary Figure 1. Channel Localisation & thresholding

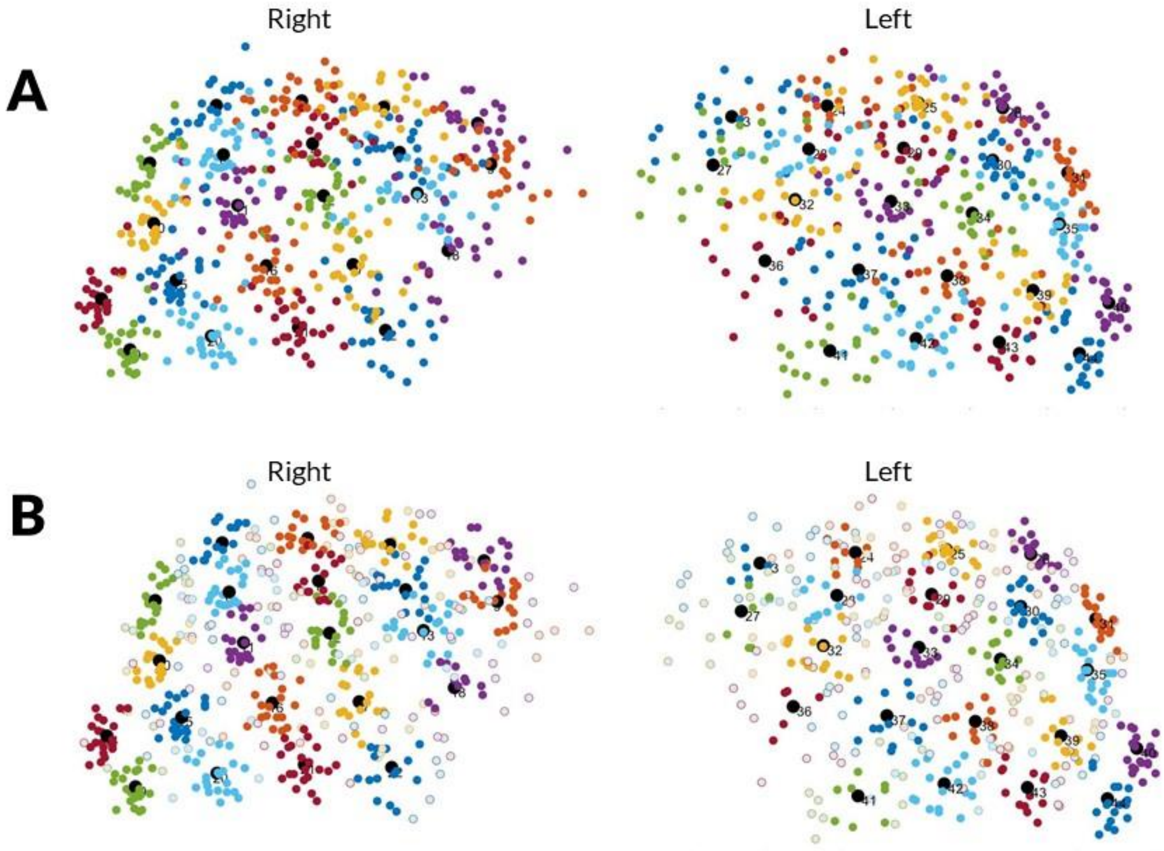

Figure S1: Channel Thresholding. A. Figure shows the canonical channel location (black) for each channel as well as the recorded position of that channel for each participant, in a different colour for each channel. **B.** Figure shows the channels that are more than 10mm away from the canonical channel location excluded (those with only the outline visible).

Supplementary results tables

Table S2. Two-way Mixed ANOVA of Follower Height.

Tests of Within-Subjects Contrasts

Source	df	F	Sig.
Watch_UnW	1	7.291	0.010
Watch_UnW * Group	1	0.089	0.767
Error(Watch_UnW)	42		
Base_Exag	1	0.266	0.609
Base_Exag * Group	1	1.017	0.319
Error(Base_Exag)	42		
Watch_UnW * Base_Exag	1	1.550	0.220
Watch_UnW * Base_Exag * Group	1	0.025	0.874
Error(Watch_UnW*Base_Exag)	42		

Tests of Between-Subjects Effects

Source	df	F	Sig.
Intercept	1	2967.865	0.000
Group	1	0.282	0.598
Error	42		

Analysis of other behavioural and physiological signals

In addition to our primary measure of follower movement height, we also analysed the Time taken per trial, conversation events during the trials, and the three physiological signals recorded by the Equivital belt, namely Heart Rate, Breathing Rate and Galvanic Skin Response. In each case, we conducted a two-way mixed ANOVA to compare the effect of trajectory, and being watched or unwatched on the dependent variable, in both NT and ASC groups. We found a significant main effect of being Watched on the time taken by the Follower [$F(1,42) = 17.72$, $p < 0.001$]. There was no main effect of Group, or of Trajectory. There was a significant interaction effect between being Watched or Unwatched and Group [$F(1,42) = 6.97$, $p = 0.012$]. A paired sample t-test showed that there was a difference in the Time taken for the ASC group to move in the Watched and Unwatched conditions [M-Watched = 22.02, SD = 7.06, M-Unwatched = 17.26, SD = 5.26; $t(21) = 4.22$, $p < 0.001$].

These results suggest that participants with ASC moved more slowly in the Watched condition. However, one reason for this could be that in the Watched condition participants had more conversations with the Leader and this lengthened trial times. We manually coded the videos of each trial to evaluate the amount of conversation between the Leader and the Follower during the Follower's turn. This was coded on a scale ranging from 1 for no conversation, to 5 where both the Leader and the Follower spoke sentences longer than two words to each other during the Follower's turn. A Pearson's product-moment correlation coefficient was calculated to assess the relationship between the time taken by the Follower and the amount of conversation. This showed that for both NT and ASC Followers there was a positive correlation between the time taken per trial and the overall conversation [$r(NT) = 0.396$, $p<0.001$; $r(ASC) = 0.604$, $p<0.001$]. This suggests that the

amount of conversation is a likely explanation for why trials took longer the Watched condition when compared with the Unwatched condition.

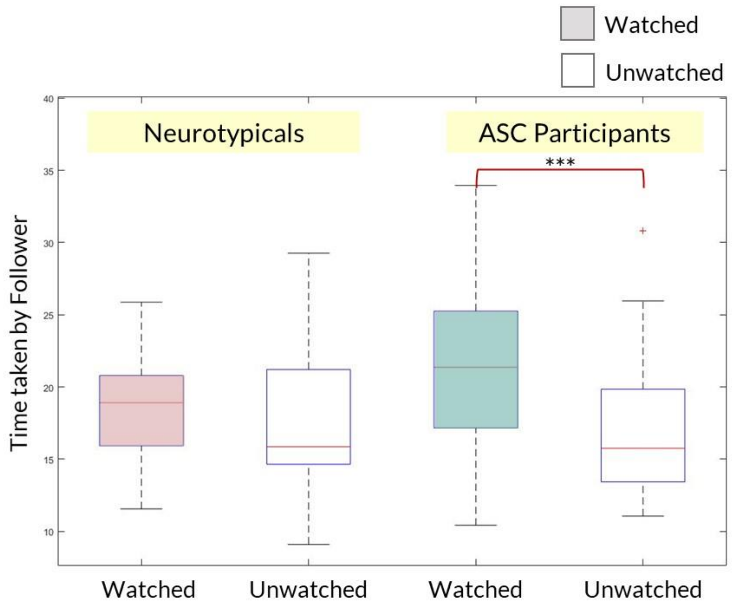

Figure S2. Two-way mixed ANOVA of Time taken by Follower. This shows the time taken by both NT and ASC groups in the Watched and Unwatched conditions.

<u>*Heart rate*</u>

We found no significant main or interaction effects of the independent variables on the heart rate of subjects.

<u>*Breathing rate*</u>

There was a significant main effect of being Watched on the Breathing rate of the Follower [$F(1,17) = 4.92$, $p = 0.041$]. There was no main effect of Group, or of Trajectory. There were no significant two-way interactions between the independent variables, but there was a significant three-way interaction effect between being Watched, Trajectory and Group [$F(1,17) = 6.28$, $p = 0.023$]. Detailed pair-wise comparisons show that both groups breathe faster in the Unwatched condition over the Watched condition, but this increase occurred in the Baseline trials for NT participants, and in the Exaggerated trials for the ASC participants. Speech patterns are once again a likely cause for the difference in breathing rates since participants are likely to breathe more rapidly when not speaking.

Again, a Pearson's product-moment correlation coefficient was calculated to assess the relationship between Follower breathing rate and the amount the Follower spoke during their turn (coded manually from the video). For the NT group we found no significant correlation, but for the ASC group we found a negative correlation between breathing rate and the amount of Follower conversation [$r(ASC) = -0.223$, $p = 0.003$] which supports our view that breathing rate was lower when the Follower was speaking more.

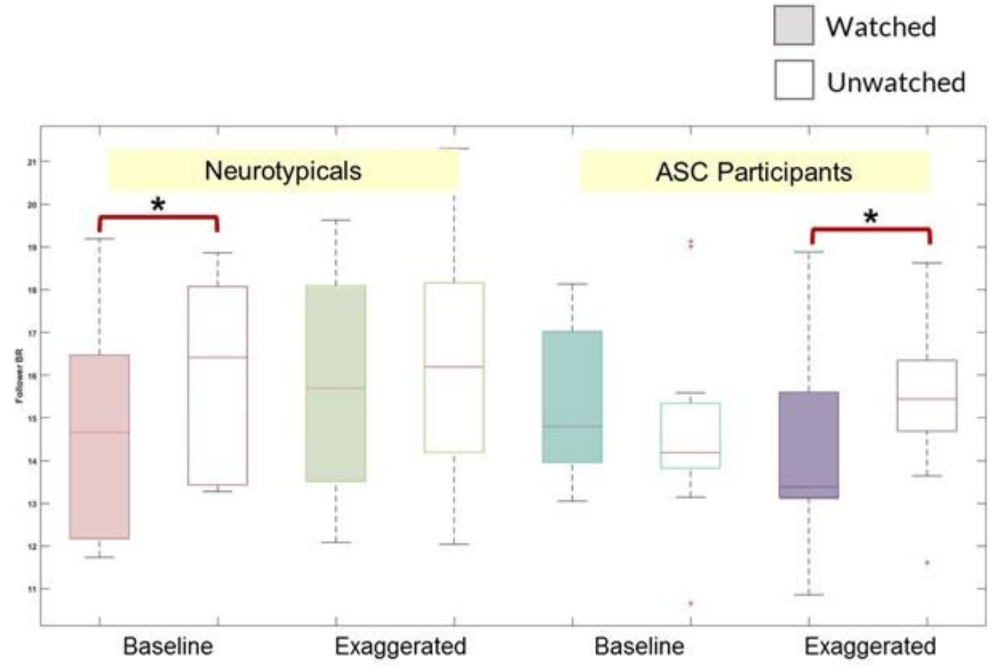

Figure S3. Two-way mixed ANOVA of Follower Breathing rate. This shows the breathing rate of both NT and ASC groups in the Baseline and Exaggerated conditions.

Galvanic skin response

There was no significant main effect of Group, Trajectory, or being Watched on the Galvanic Skin Response of the Follower. There was a two-way interaction effect between being Watched and trajectory [F(1,27) = 5.62, p = 0.03]. There was no three-way interaction effect.

Table S3. Two-way Mixed ANOVA of Follower Galvanic Skin Response.

Tests of Within-Subjects Contrasts

Source	df	F	Sig.
Watch_UnW	1	0.59	0.449
Watch_UnW * Group	1	3.33	0.079
Error(Watch_UnW)	27		
Base_Exag	1	0.79	0.382
Base_Exag * Group	1	1.78	0.193
Error(Base_Exag)	27		
Watch_UnW * Base_Exag	1	5.62	0.025
Watch_UnW * Base_Exag * Group	1	0.23	0.637
Error(Watch_UnW*Base_Exag)	27		

Tests of Between-Subjects Effects

Source	df	F	Sig.
Intercept	1	103.53	0.000
Group	1	2.49	0.126
Error	27		

Despite the two-way interaction, however, detailed pair-wise t-tests do not offer any support for a difference in GSR between conditions. A paired-sample t-test of the GSR in the watched and unwatched condition for baseline trials [t(28) = 0.73, p = 0.47] and for exaggerated trials [t(28) = -0.172, p = 0.10] showed no statistical significance. Perhaps with a larger sample size in future studies this would be worthy of exploring further. At present there is no support here for a difference in arousal between conditions.

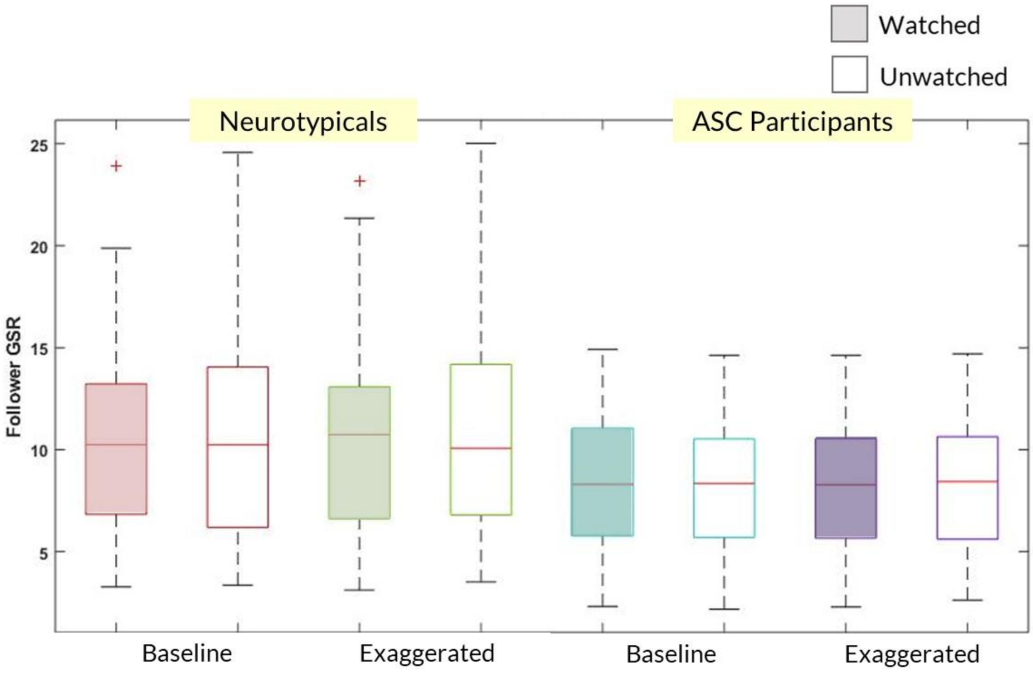

Figure S4. Two-way mixed ANOVA of Follower Galvanic skin response. This shows the Galvanic skin response of both NT and ASC groups in the Baseline and Exaggerated conditions.
